# Optimized Production of Biodiesel from Waste Cooking Oil by Lipase Immobilized on Magnetic Nanoparticles

**DOI:** 10.3390/ijms141224074

**Published:** 2013-12-11

**Authors:** Chi-Yang Yu, Liang-Yu Huang, I-Ching Kuan, Shiow-Ling Lee

**Affiliations:** Department of Bioengineering, Tatung University, 40 Zhongshan N. Rd. Sec. 3, Taipei 10452, Taiwan; E-Mails: chrisyu@ttu.edu.tw (C.-Y.Y.); cherryfish1115@gmail.com (L.-Y.H.); iching@ttu.edu.tw (I.-C.K.)

**Keywords:** biodiesel, lipase, magnetic nanoparticles, response surface methodology, waste cooking oil

## Abstract

Biodiesel, a non-toxic and biodegradable fuel, has recently become a major source of renewable alternative fuels. Utilization of lipase as a biocatalyst to produce biodiesel has advantages over common alkaline catalysts such as mild reaction conditions, easy product separation, and use of waste cooking oil as raw material. In this study, *Pseudomonas cepacia* lipase immobilized onto magnetic nanoparticles (MNP) was used for biodiesel production from waste cooking oil. The optimal dosage of lipase-bound MNP was 40% (*w*/*w* of oil) and there was little difference between stepwise addition of methanol at 12 h- and 24 h-intervals. Reaction temperature, substrate molar ratio (methanol/oil), and water content (*w*/*w* of oil) were optimized using response surface methodology (RSM). The optimal reaction conditions were 44.2 °C, substrate molar ratio of 5.2, and water content of 12.5%. The predicted and experimental molar conversions of fatty acid methyl esters (FAME) were 80% and 79%, respectively.

## 1. Introduction

Biodiesel is defined as the fatty acid alkyl monoesters derived from renewable feedstocks such as vegetable oils and animal fats [1]. It draws much attention as an alternative fuel because it is biodegradable, non-toxic, and can be used directly or blended with conventional petrodiesel in unmodified diesel engines. As compared to petrodiesel, biodiesel has a higher cetane number, no aromatics, almost no sulfur, and contains 10%–11% oxygen by weight [2], thus reducing the emission of carbon monoxide, hydrocarbon, and particulate matter in the exhaust gas. Biodiesel is commonly produced by transesterification of virgin vegetable oils with short chain alcohols using alkaline catalysts. However, the process requires high quality food-grade vegetable oils with low level of free fatty acids (FFA) to avoid saponification, which leads to low biodiesel conversion and causes difficulties in the separation of glycerol. A major hurdle in the commercialization of biodiesel is its high manufacturing cost, primarily from virgin vegetable oils. Therefore, waste cooking oil (WCO) has become a promising feedstock for biodiesel production. WCO is much less expensive than pure vegetable oils from soybean, sunflower or canola, and it is currently used as animal feed or is simply discarded. However, the harmful compounds of WCO may return into the food chain when used as animal feed and the disposal of WCO often leads to contamination of recipient waters [3]. In addition to the advantage of lower cost, utilization of WCO as a feedstock for biodiesel production partly solves the problem of disposing WCO. Biodiesel attained out of animal fat and WCO has a lower price than those derived from refined vegetable oils and fossil diesel [4].

Lipases (E.C.3.1.1.3) are capable of catalyzing a variety of reactions such as hydrolysis, alcoholysis, esterification, transesterification, and hence are widely used in industry [5]. Biodiesel can also be synthesized via lipase-catalyzed transesterification; the process produces high purity products and enables easy separation of the glycerol byproduct [6]. The enzymatic process is compatible with low quality feedstocks with high levels of FFA. It also requires less energy input due to lower reaction temperature than the akali-catalyzed process. However, there has been very limited commercial success due to the high cost of lipases. One common strategy for reducing the cost of lipases is to recycle the biocatalyst through immobilization [7].

Various immobilization techniques have been applied to lipases for the production of biodiesel. Support materials such as Celite [8], acrylic resin [9], and ion exchange resins [10,11] were used for attaching lipases through adsorption. The adsorption technique is easy to perform, but it suffers from desorption of enzyme molecules. Immobilization through covalent bonds has the advantage of minimal enzyme leakage, and support like electrospun polyacrylonitrile was reported [12]. Cross-linked enzyme aggregates can be formed by means of bifunctional or multifunctional reagents such as glutaraldehyde [13]. Lipases can be also entrapped in polymeric matrix like phyllosilicate sol-gel [14] and silica gels [15] or encapsulated in silica aerogel [16].

Magnetic nanoparticles (MNP) has been widely applied in many fields of life science, for instance, magnetic resonance imaging contrast enhancement, tissue repair, immunoassay, hyperthermia, and drug delivery [17], owing to possessing unique properties of nontoxicity, biocompatibility, injectability, and high level of accumulation in the target tissue and organ [18]. It has been used as carriers for immobilizing drugs, proteins, enzymes, antibodies, and nucleotides [17]. Use of MNP as support for enzyme immobilization allows for large surface area for potential high enzyme loading, selective separation from the reaction mixture under magnetic field, and low mass transfer resistance due to small particle size [19]. We have previously immobilized lipase from *Pseudomonas cepacia* onto MNP for the synthesis of fatty acid methyl esters (FAME) with soybean oil as a feedstock [20,21].

Response surface methodology (RSM), a collection of mathematical and statistical techniques useful for modeling and analysis of problems in which a response of interest is influenced by several variables [22], has been widely applied to the synthesis of biodiesel. For instance, temperature, substrate molar ratio, and *n*-hexane content were optimized for the production of biodiesel using lipase entrapped in biomimetic silica [23], or temperature, flow rate, and substrate molar ratio were optimized for continuous production of biodiesel in a lipase-catalyzed packed-bed reactor [9,24].

The production of biodiesel using immobilized lipase has been studied extensively; however, most of related studies utilized pure vegetable oils as feedstocks [7]. In this work, we evaluated the potential of WCO as a feedstock for the synthesis of FAME using lipase immobilized on MNP as a catalyst. The crucial reaction variables (temperature, substrate molar ratio, and water content) were optimized with RSM. In addition, reusability and storage stability of immobilized enzyme were also evaluated.

## 2. Results and Discussion

### 2.1. Effects of Amount of Added Lipase on Immobilization Efficiency and Activity Recovery

**Figure 1.**
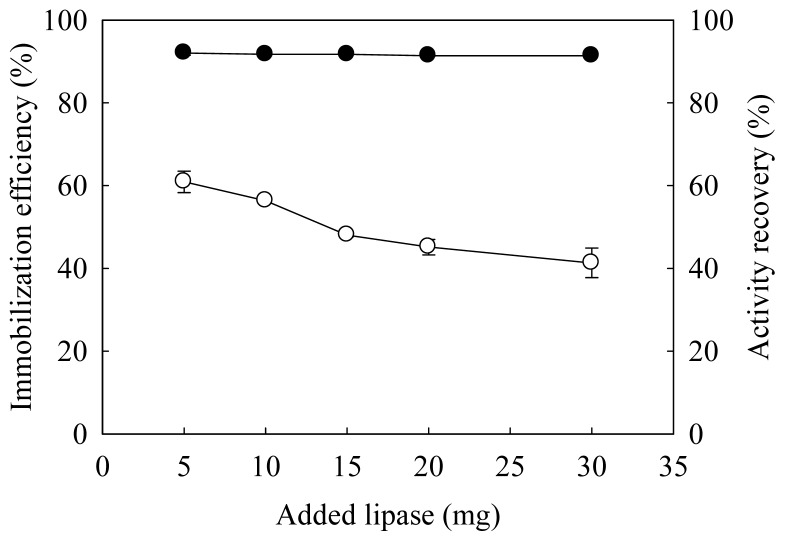
Effects of amount of lipase added during immobilization on immobilization efficiency (●) and activity recovery (○).

Effects of amount of added lipase on immobilization efficiency (ratio of amount of immobilized to added lipase) and activity recovery (ratio of specific activities for immobilized to free lipase) are shown in Figure 1. Maximal activity recovery of 60% with the corresponding immobilization efficiency of 92.3% was observed when 5 mg of lipase was added. The activity recovery decreased as the amount of lipase increased; however, the immobilization efficiency showed little change. The decrease in activity recovery could be attributed to greater steric hindrance generated and less accessible active sites as more enzyme molecules were attached to the support.

We also evaluated the external mass transfer resistance with Mears’ criterion [25]:

(1) $$\frac{-r_{A}^{\prime }\rho_{\text{b}}Rn}{k_{\text{c}}C_{A\text{b}}}<0.15$$

where $-r_{A}^{\prime }$ is the rate of reaction; *ρ*_b_ is the bulk density of catalyst; *R* is the radius of catalyst particle; *n* is the reaction order; *k*_c_ is the mass transfer coefficient; *C_A_*_b_ is the bulk concentration of the substrate. External mass transfer resistance can be neglected if Inequality (1) is satisfied. The value of Mears’ criterion for lipase-bound MNP was 3.8 × 10^−3^ when calculated with $-r_{A}^{\prime }=0.02\;\text{kmol}/(\text{kg catalysts})$, *ρ*_b_ = 5.15 × 10^3^ kg/m^3^ for magnetite [26], *R* = 8 × 10^−9^ m, *n* = 1, *k*_c_ = 0.29 m/s, and *C_A_*_b_ = 0.74 × 10^−3^ kmol/m^3^. The result was significantly smaller than the critical value of 0.15, indicating that external mass transfer resistance can be neglected. The *k*_c_ was estimated with the correlation proposed for free convection around a solid sphere [27]. The diffusion coefficient of *p*-nitrophenyl palmitate substrate, 2.3 × 10^−5^ cm^2^/s, required for the determination of *k*_c_ was determined using the modified Stokes-Einstein equation [28]. The actual reaction order (*n*) was between 0 and 1 because the substrate concentration of 0.74 mM was close to the *K_m_* value of 1.5 mM for immobilized lipase [20], and hence the Mears’ criterion was over-estimated with the assumption of *n* = 1. As more lipase was added, the radius of the lipase-bound MNP was increased with the attachment of more lipase to MNP and a porous outer layer of lipase may form. The external mass transfer resistance, however, was not limiting because the calculated Mears’ criterion was much smaller than 0.15 even if the radius was increased by several folds. Nevertheless, the formation of an outer layer of lipase possibly caused a decrease in the effective diffusion coefficient within the layer [29]. The internal mass transfer may become limiting. Because the characteristics of the lipase layer are not known, the extent of internal mass transfer resistance is difficult to estimate.

### 2.2. Effects of Dosage of Lipase-Bound MNP and Stepwise Addition of Methanol on the Conversion of FAME

**Figure 2.**
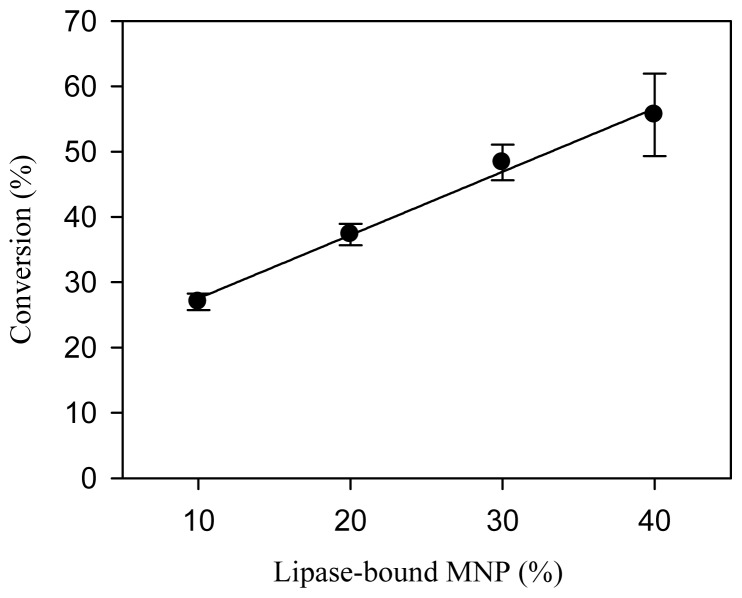
Effects of dosage of lipase-bound magnetic nanoparticles (MNP) on the conversion of fatty acid methyl esters (FAME). The dosage was expressed in weight percentage of waste cooking oil. The reaction was carried out at 40 °C for 72 h with water content of 10% (*w*/*w* of oil). The molar ratio of methanol to oil was 3:1; three separate additions at 0, 24 and 48 h, one third of the total amount each time. The equation of the fitted line is *y* = *x* + 17.8 with *R*^2^ = 0.99.

As shown in Figure 2, the conversion of FAME increased linearly with dosage of lipase-bound MNP in the range examined. Maximal FAME conversion of 55.6% was observed with 40% (*w*/*w* of oil) lipase-bound MNP, which was then used in later experiments.

**Figure 3.**
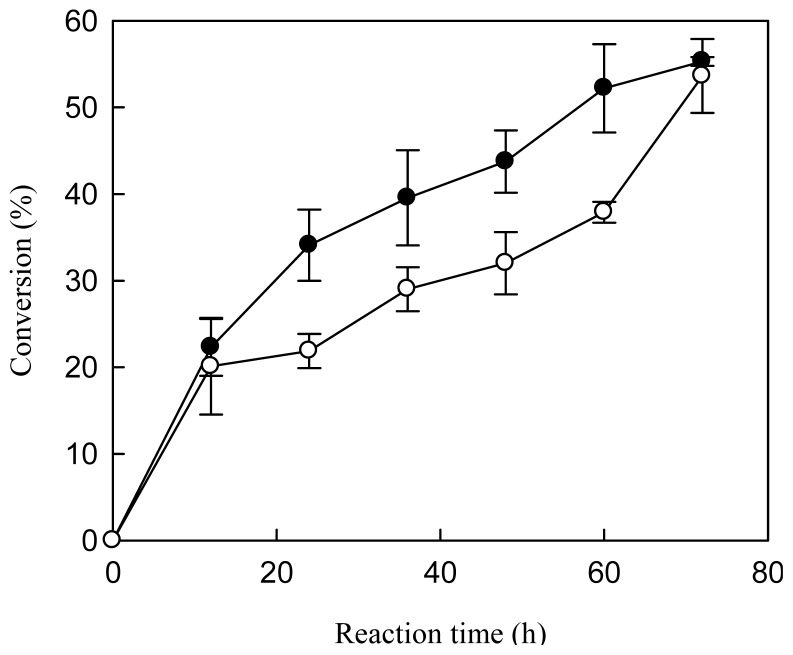
Time course of the conversion of FAME when methanol was added at different intervals. The reaction was carried out at 40 °C for 72 h with water content of 10% (*w*/*w* of oil). The molar ratio of methanol to oil was 3:1; three separate additions at 0, 12 and 24 h (●) or at 0, 24 and 48 h (○), one third of the total amount each time.

Alcoholysis with relatively long-chain and branched alcohols proceeds efficiently even in organic-solvent free systems, but not in the case of methanolysis due to the inactivation of lipase by methanol [30]. One common strategy to avoid such inhibition is to add methanol in a stepwise fashion [30,31]. We examined the effect of stepwise addition at 0, 12 and 24 h or 0, 24 and 48 h (Figure 3). Stepwise addition at 12 h-intervals resulted in a higher rate of FAME conversion in comparison with the 24 h-interval addition. However, the conversions after 72 h were similar regardless of the interval used. For later experiments, stepwise addition of methanol at 24 h-intervals was selected.

### 2.3. Model Fitting and Analysis of Variance

In addition to temperature and amount of methanol, the amount of water is also critical for the synthesis of FAME. Lipase possesses the unique feature of acting at the interface between an organic and an aqueous phase. The addition of water facilitates the formation of interfacial area; however, excess water may stimulate competitive hydrolysis reactions [7]. The optimal water content is a compromise between minimizing hydrolysis and maximizing enzyme activity for the transesterification reaction [15].

Based on previous reports using *P. cepacia* lipase immobilized on various supports for the transesterification of triglyceride to biodiesel [15,32,33], the variables selected for optimization and the corresponding ranges were temperature from 35 to 50 °C, water content of 1% to 20% (*w*/*w* of oil), and the molar ratio of methanol to oil from 3:1 to 8:1. The design of experiments and the corresponding data are given in Table 1. After fitting the data with various models followed by analysis of variance (ANOVA), the following quadratic polynomial most suitably described the correlation between conversion and the tested variables:

(2) $$Y=-347.13+13.1A+33.48B+8.38C-0.16A^{2}-3.31B^{2}-0.43C^{2}+0.06AB+0.07AC-0.1BC$$

where *Y*, *A*, *B* and *C* were conversion of FAME, temperature, substrate molar ratio (methanol/oil), and water content (%, *w*/*w* of oil), respectively. The *F*-value of 25.92 for the model was higher than *F*_0.01,9,7_ of 6.72, indicating the model was significant at confidence level of 99%. The *F*-value for lack of fit was 6.42, much lower than *F*_0.01,3,4_ of 16.69, indicating lack of fit was insignificant. Overall, the model had a small *p*-value of 0.0001 and a suitable coefficient of determination (*R*^2^ = 0.97), clearly indicating that the model was highly significant and sufficient to describe the correlation between the conversion of FAME and the tested variables. The high value of adjusted determination coefficient (Adj. *R*^2^ = 0.93) also supported the significance of the model. The value of adequate precision (a measure of signal to noise ratio) of the model was 14.29, which is greater than four, thus providing adequate model discrimination [22]. Water content and all the square terms were significant for the process with *p*-values smaller than 0.05 (Table 2).

### 2.4. Effects of Variables and Their Optimization

**Figure 4.**
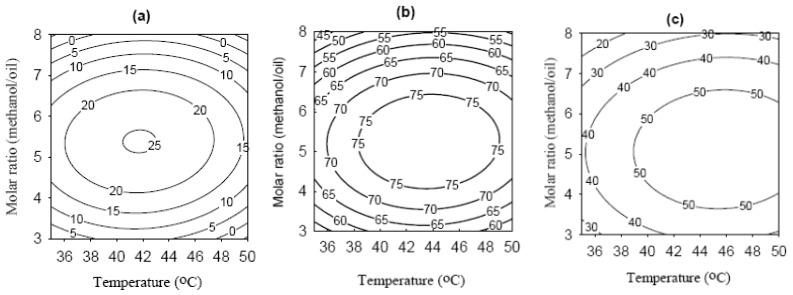
Contour plots of the molar conversion of FAME at different water content. (**a**) 1%; (**b**) 10.5%; and (**c**) 20%. The water content was expressed in weight percentage of waste cooking oil.

The correlation between the conversion of FAME and tested variables can be better understood by examining the contour plots. In Figure 4, the conversion increased significantly when water content increased from 1% to 10.5% (Figure 4a*vs.* 4b), suggesting that certain amount of water was required to activate the immobilized lipase. Nevertheless, when increasing the water content further to 20%, the conversion decreased, indicating that the hydrolysis may start to compete with methanolysis. As the temperature was elevated from 35 to around 42.5 °C (center point), the conversion increased. However, when increasing the temperature further to 50 °C, the conversion started to decrease, showing that part of the immobilized lipase may start to inactivate. The conversion increased with the substrate molar ratio up to 5–6, but decreased when increasing the substrate molar ratio up to 8, which could be explained by the inactivation of lipase caused by excessive methanol.

The optimal reaction conditions were 44.2 °C, substrate molar ratio of 5.2, and water content of 12.5%; the predicted and experimental values of conversion were 80% and 79%, respectively. The optimized conversion of FAME was higher than those using lipase from *Thermomyces lanuginosa* or *Candida antarctica* immobilized on granulated silica or *C. antarctica* lipase immobilized on macroporous acrylic resin (SP435) with restaurant grease as a feedstock [34]; but lower than those using *P. cepacia* lipase entrapped within a phyllosilictae sol-gel matrix (PS-30) with tallow and grease as feedstocks [14,34]. Our previous study using soybean oil as a feedstock showed 93% conversion of FAME with the same preparation of lipase-bound MNP [20]. The lower conversion with WCO could be explained by the presence of oxidized compounds such as aldehydes, epoxides, and polymers which were unrecognized as substrates by lipase [3].

### 2.5. Storage Stability and Reusability of Immobilized Lipase

**Figure 5.**
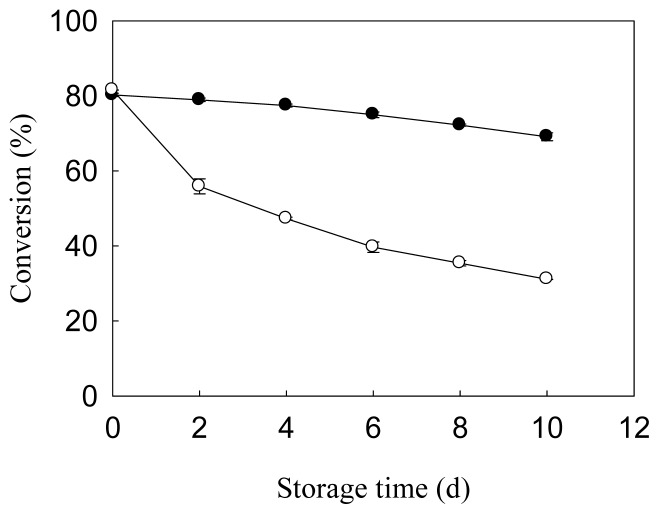
Stability of *Pseudomonas cepacia* lipase immobilized on magnetic nanoparticles after stored at 4 °C (●) and room temperature (○) at the time indicated. 40% (*w*/*w* of oil) immobilized lipase was used to catalyze transesterification using 4.8 g waste cooking oil under optimal reaction conditions for 72 h.

The storage stability of immobilized lipase at 4 °C and room temperature was examined (Figure 5). Immobilized lipase stored at room temperature decayed at a faster rate as compared to that stored at 4 °C. The conversion of FAME for lipase stored at room temperature and 4 °C after 10 days were 31.1% and 69.1%, respectively, clearly indicating better storage stability at 4 °C.

**Figure 6.**
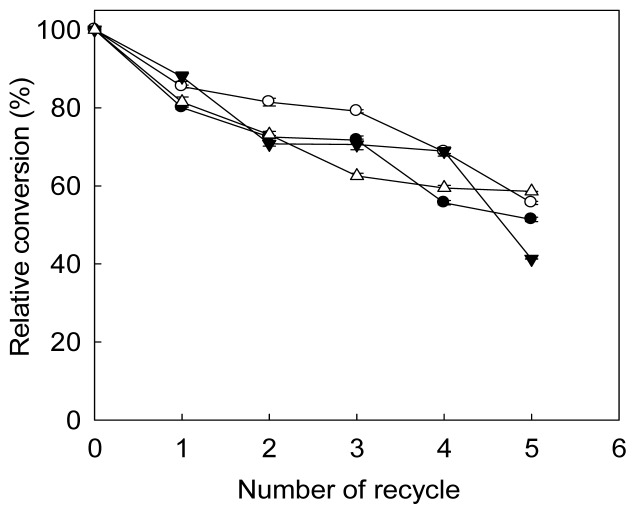
Reusability of *Pseudomonas cepacia* lipase immobilized on magnetic nanoparticles. Immobilized lipase was recycled without washing (▼) or after washing with *tert*-butanol (○); *n*-hexane (△); and deionized water (●). The initial conversion was defined as 100%. 40% (*w*/*w* of oil) immobilized lipase was used to catalyze transesterification using 4.8 g waste cooking oil under optimal reaction conditions for 72 h.

The reusability of immobilized lipase after washing with different solvent is shown in Figure 6. After three repeated uses, immobilized lipase recycled by washing with *tert*-butanol retained most of its initial conversion. *tert*-Butanol was reported being effective in the regeneration of immobilized lipase [35], perhaps due to its ability to alleviate the negative effects of both methanol and glycerol on activity [36]. After five cycles, lipase recycled without washing had the lowest relative conversion; however, the conversions showed little difference regardless of the solvent used. The decrease in FAME conversion after recycling can be partially attributed to the loss of lipase-bound MNP. In our previous work, lipase-bound MNP exhibited 89% of the initial activity after incubation at 40 °C for 30 min [20]. This implicated that thermal inactivation of immobilized lipase also contributed to the decrease in the conversion of FAME during reuse.

## 3. Experimental Section

### 3.1. Preparation of MNP

All reagents were purchased from Wako (Osaka, Japan) unless otherwise specified. MNP was prepared by dissolving 0.4 g of FeCl_2_·4H_2_O and 1.08 g of FeCl_3_·6H_2_O in 20 mL deionized water (final concentrations of Fe^2+^ and Fe^3+^ were 0.1 and 0.2 M, respectively), followed by addition of 15 mL of 29% (*v*/*v*) NH_4_OH under vigorous stirring at room temperature. The precipitate was heated at 80 °C for 30 min before washing with 40 mL of deionized water twice followed by 40 mL of ethanol twice. The precipitate was finally resuspended in 40 mL of deionized water and then lyophilized. The untreated MNP were close to spherical with an average diameter of 16 nm by examining with high resolution TEM (JEOL, Akishima, Japan), and the XRD (MAC Science, Yokohama, Japan) pattern confirmed the synthesized MNP was pure Fe_3_O_4_ with a spinel structure [20].

### 3.2. Immobilization of Lipase

The procedure used was the same as previous report with minor modifications [19]. One hundred and fifty milligrams of MNP was added to 10 mL of binding buffer (3 mM sodium phosphate buffer, pH 6, containing 0.1 M NaCl) followed by sonication for 10 min. After removing the binding buffer, MNP was activated with 10 mL of 18.75 mg/mL carbodiimide prepared in the binding buffer for 15 min under sonication. MNP was then washed with 10 mL binding buffer three times, followed by incubation with 10 mL of 0.5 to 3 mg/mL Amano lipase PS (from *P. cepacia*; Sigma-Aldrich, St. Louis, MO, USA) solution prepared in the binding buffer at 4 °C for 30 min under sonication. After separation with a magnet, the lipase-bound MNP was washed with binding buffer several times and ready for use. The residual protein concentration in the supernatant was determined with BCA assay [37]. The immobilization efficiency was defined as follows:

(3) Immobilization efficiency (%)=[(amount of added lipase-residual lipase in the supernatant)/amount of added lipase]×100

### 3.3. Assay for Lipase Activity

The assay was modified from that described by Pencreac’h *et al.* [38]. The assay mixture contained 90 μL of 8.25 mM *p*-nitrophenyl palmitate (Sigma-Aldrich) in isopropanol and 810 μL of 50 mM Tris-HCl, pH 8.0, with 0.5% (*w*/*v*) Triton X-100 and 0.12% (*w*/*v*) arabic gum preheated to 40 °C. To initiate the reaction, 100 μL of lipase solution or suspension of immobilized lipase diluted to appropriate concentration with 1% (*w*/*v*) bovine serum albumin was added. The change in absorbance at 410 nm was monitored for 5 min at 40 °C using a thermostated spectrophotometer (UV-1800, Shimadzu, Kyoto, Japan). The activity was calculated from the difference in absorbance between 2 and 5 min with a standard curve for the hydrolysis product, *p*-nitrophenol. One activity unit was defined as the production of 1 μmol of *p*-nitrophenol per min at 40 °C. Measurements were performed in triplicates.

### 3.4. Experimental Design

Although the central composite design is often used for fitting second-order models, the Box-Behnken design is also efficient and needs less number of runs [22]. In our study, a 3-level-3-factor Box-Behnken design with five replicates at the center was applied and required 17 experiments. The variables selected for the synthesis of biodiesel and the corresponding ranges were reaction temperature (35–50 °C), substrate molar ratio (methanol: oil = 3:1–8:1), and water content (1%–20%, *w*/*w* of oil). Levels of the factors, in terms of coded and uncoded forms, are shown in Table 1. Treatments were performed in a fully random order to avoid bias.

### 3.5. Transesterification of Oil to Biodiesel

In a typical reaction, 1.92 g of lyophilized lipase-bound MNP (40%, *w*/*w* of oil) and 4.8 g of waste cooking oil were mixed in a 25 mL Erlenmeyer flask, followed by three separate additions of methanol, at 0, 24 and 48 h with one third of the total amount (methanol:oil = 3:1–8:1) each time. The mixture also contained deionized water (1%–20%, *w*/*w* of oil). The reaction mixture was incubated in the temperature range of 35–50 °C using a water bath with orbital shaking at 200 rpm for 72 h.

### 3.6. Analysis of FAME

The oil phase of the reaction mixture was first treated with 30 mg of sodium sulfate followed by centrifugation at 7500× *g* for 5 min. Fifty microliters of treated sample was mixed with 1 mL of 10 mg/mL methyl heptadecanoate (TCI, Portland, OR, USA) in hexane as an internal standard. The conversion of FAME was determined by injecting 1 μL of the sample into a gas chromatograph (Shimadzu GC-14A, Kyoto, Japan) equipped with a flame-ionization detector (FID, Shimadzu). A BPX70 capillary column (30 m × 0.25 mm i.d.; SGE Analytical Science, Ringwood, Australia) with hydrogen as carrier gas at a constant pressure of 6 kg/cm^2^ was used. The injector and FID temperatures were set at 250 and 280 °C, respectively. The oven temperature was initially held at 150 °C for 30 s and then increased to 180 °C at 10 °C/min, finally to 198 °C at 1.5 °C/min. The amount of FAME in sample was determined from standard curves and measurements were performed in triplicates. The conversion was defined as follows:

(4) $$\mathrm{Conversion}\;(\%)=\frac{moles\;of\;FAME\;produced}{3\times moles\;of\;oil}\times 100$$

### 3.7. Data Analysis

The experimental data (Table 1) were fitted to the following second-order polynomial equation using the analysis procedure of the Design Expert software version 6.01 (Stat-Ease, Minneapolis, MN, USA):

(5) $$Y=\beta_{0}+\sum_{i=1}^{3}\beta_{i}x_{i}+\sum_{i=1}^{3}\beta_{ii}x_{i}^{2}+\sum_{i=1}^{2}\sum_{j=i+1}^{3}\beta_{ij}x_{i}x_{j}$$

where *Y* is response (conversion of FAME); *β*_0_, *β_i_*, *β_ii_*, and *β_ij_* are constant coefficients; and *x_i_* and *x_j_* are the uncoded independent variables. All analytical steps including analysis of variance (ANOVA), regression analysis, optimization of the variables, and plotting of response surfaces were performed using the same software.

## 4. Conclusions

In this work, we demonstrated the potential of *P. cepacia* lipase immobilized on MNP as a biocatalyst for the synthesis of FAME using WCO as a feedstock, and the conversion of FAME reached 79% under optimal reaction conditions, which was comparable to those using other lipases in immobilized form. The proposed process may lower the production cost of biodiesel and facilitate the disposal of WCO. The immobilized lipase exhibited good storage stability at 4 °C and can be easily recovered by magnetic field for repeated use. Approximately 80% of the initial FAME conversion was retained after three repeated uses when lipase-bound MNP was washed with *tert*-butanol. Nevertheless, the reusability and storage stability at room temperature require further improvement for the immobilized lipase to be practical for industrial applications. Thermal inactivation is critical for both reusability and storage stability. One possible approach for improvement is to use thermally stable lipases [39,40]. Because large amount of lipase-bound MNP was used for the transesterification, those away from the magnetic field were easily washed off during recycling. Such loss of the biocatalyst could be reduced if stronger magnetic field is applied. Alternatively, the loss of lipase-bound MNP during recycling could be improved by using a packed-bed reactor, which also allows for continuous removal of products and protection of the enzyme from mechanical shear.

## Figures and Tables

**Table 1.** Three-level-three-factor Box-Behnken design of experiments and the corresponding conversions.

Treatment No. a	Variable b			Conversion (%)

	Temperature (°C)	Molar ratio (methanol/oil)	Water content (%, *w*/*w* of oil)	
1	35 (−1)	3 (−1)	10.5 (0)	48 ± 1
2	50 (1)	3 (−1)	10.5 (0)	54.1 ± 0.3
3	35 (−1)	8 (1)	10.5 (0)	42.70 ± 0.01
4	50 (1)	8 (1)	10.5 (0)	52.6 ± 0.4
5	35 (−1)	5.5 (0)	1 (−1)	13.5 ± 0.1
6	50 (1)	5.5 (0)	1 (−1)	8 ± 1
7	35 (−1)	5.5 (0)	20 (1)	44.6 ± 0.9
8	50 (1)	5.5 (0)	20 (1)	58.13 ± 0.06
9	42.5 (0)	3 (−1)	1 (−1)	14.0 ± 0.9
10	42.5 (0)	8 (1)	1 (−1)	5.49 ± 0.02
11	42.5 (0)	3 (−1)	20 (1)	38.2 ± 0.5
12	42.5 (0)	8 (1)	20 (1)	20.0 ± 0.9
13	42.5 (0)	5.5 (0)	10.5 (0)	75.1 ± 0.8
14	42.5 (0)	5.5 (0)	10.5 (0)	76 ± 1
15	42.5 (0)	5.5 (0)	10.5 (0)	79.0 ± 0.2
16	42.5 (0)	5.5 (0)	10.5 (0)	82.1 ± 0.3
17	42.5 (0)	5.5 (0)	10.5 (0)	84.0 ± 0.8

a The treatments were performed in random order;

b The values of 1, −1 and 0 in parentheses were coded levels.

**Table 2.** Analysis of variance (ANOVA) for the regression model and respective model terms.

Source	Sum of squares	Degree of freedom	Mean square	*F*-value	Prob > *F*^a^,^b^
Model	11398.83	9	1266.537	25.92057	0.0001
Temperature (A)	69.32531	1	69.32531	1.418791	0.2724
Substrate molar ratio (B)	142.1298	1	142.1298	2.908786	0.1319
Water content (C)	1799.7	1	1799.7	36.83213	0.0005
*A*^2^	346.665	1	346.665	7.094744	0.0323
*B*^2^	1805.258	1	1805.258	36.94588	0.0005
*C*^2^	6430.1	1	6430.1	131.5965	<0.0001
*AB*	4.2849	1	4.2849	0.087693	0.7757
*AC*	91.48922	1	91.48922	1.872391	0.2135
*BC*	23.3289	1	23.3289	0.477442	0.5118
Residual	342.0356	7	48.86223		
Lack of fit	283.2356	3	94.41188	6.422577	0.0521
Pure error	58.8	4	14.7		
Cor total	11740.87	16			

a Significant at “Prob > *F*” lower than 0.05;

b Insignificant at “Prob > *F*” higher than 0.1.
